# Sunscreen Use among Medical Undergraduate Students in a Medical College: A Descriptive Cross-sectional Study

**DOI:** 10.31729/jnma.6417

**Published:** 2022-01-31

**Authors:** Kumud Chapagain, Gajendra Prasad Rauniar

**Affiliations:** 1 Department of Clinical Pharmacology & Therapeutics, B.P. Koirala Institute of Health Sciences, Dharan, Nepal

**Keywords:** *medical students*, *sun exposure*, *sun protection*, *sunscreen*

## Abstract

**Introduction:**

Unprotected sun exposure could cause tanning, burning, hyperpigmentation, skin aging, and even skin cancer. Regular sunscreen use is a safeguard and a primary preventive strategy against sunlight-related damages. This study aims to find out the prevalence of sunscreen use among medical undergraduate students of a medical college of Eastern Nepal.

**Methods:**

A descriptive cross-sectional study was conducted among the current medical undergraduate students of 2 medical colleges in Nepal on sunscreen use from September 2020 to October 2020. Convenience sampling was done to reach the sample size of 300. Data collection was done after taking ethical approval from the Institutional Review Committee (IRC/1778/020). Data analysis was done by using Statistical Package for the Social Sciences version 11.5 software. Point estimate at 95% Confidence Interval was calculated along with frequency and proportion for binary data.

**Results:**

The prevalence of sunscreen users was found to be 227 (75.67%) (70.81-80.52 at 95% Confidence Interval) among whom only 35 (15.41%) were regular users. Majority 144 (63.43%) applied sunscreen only on the face, 72 (31.71%) considered reapplication, and 155 (68.2%) knew the Sun Protection Factor value of their sunscreen. Cosmetic sunscreen was considered by the majority of the users 151 (66.51%). Greasy feeling 73 (100%) was the reason for avoiding sunscreen use amongst the non-users 73 (24.34%).

**Conclusions:**

The prevalence of sunscreen users among medical undergraduate students from our study was higher than other studies conducted in similar settings.

## INTRODUCTION

Skin damage and many skin diseases are some of the unavoidable consequences of excessive unprotected sun exposure. Short-term Ultra Violet (UV) exposure leads to acute damage including burning and tanning and long-term exposure causes chronic skin damage such as hyperpigmentation, skin aging, and skin cancer (melanoma and non-melanoma skin cancer).^1^

Despite sunny hot weather, in Asian and African countries, the incidence of skin cancer is much lower than among the Caucasians,^2^ but in a tropical and developing country like Nepal inflammatory disease is very common.^3^ Regular sunscreen use reduces the transmission of UV radiation and is a safeguard and a primary preventive strategy against these sunlight- related damages.^4^

This study aims to find out the prevalence of regular sunscreen use among medical undergraduate students of a medical college of Eastern Nepal.

## METHODS

A descriptive cross-sectional study was conducted among the current medical undergraduate students of B. P. Koirala Institute of Health Sciences (BPKIHS), Dharan, Nepal from 1^st^ September to 15^th^ October 2020. Ethical approval was obtained from the Institutional Review Committee of BPKIHS (IRC/1778/020). Prior to the study, each student's email address was retrieved from the phase coordinator office and an individual email with the information sheet, the consent form and the Google link of the questionnaire was sent to them via their email address. Inclusion criteria constituted all the medical students from first year to fourth year. Students who did not agree to participate in the study or failed to give informed consent were excluded from the study. Confidentiality of the participants was maintained. Regular use of sunscreen was defined as using sunscreen always or most of the time when outside on a warm, sunny day for more than an hour.

The sample size was calculated using the formula,

n = Z^2^ × (p × q) / e^2^

  = (1.96)^2^ × 0.5 × (1-0.5) / (0.05)^2^

  = 385

Where,

n = required sample size
Z= 1.96 at 95% Confidence Interval (CI)
p= prevalence of sunscreen users among medical graduates taken as 50% for maximum sample size calculation
q= 1-p
e= margin of error, 5%

Using the formula for finite population correction, n = n/(1+ n/N)

The corrected sample size of the source population of 572 students was

n = 385 / (1+385/572)

  = 229.27

  ~ 330

After considering a 20% non-response rate, the final sample size was 276. However, the study was conducted among 300 students. Convenience sampling technique was used.

A pretested, structured, preformed online questionnaire was used for data collection, which was finalized after pretesting on 30 students and amended accordingly. The questionnaire mainly focused on demographics and self-reported skin type (Fitzpatrick skin type),^5^ the knowledge and practice related to sun exposure, and regular sunscreen use. Among 10 questions, 9 questions were related to knowledge, and 10 questions were related to practice.

The Google link form was closed for receiving the responses after the adequate sample was achieved. Microsoft Excel 2010 was used for data entry and Statistical Package for the Social Sciences version 11.5 was used for the analysis of data. Descriptive statistics such as frequency, mean, standard deviation, and percent were calculated to describe the characteristics of the sample. Point estimate at 95% Confidence Interval was calculated along with frequency and proportion for binary data.

## RESULTS

The prevalence of sunscreen users among medical undergraduate students from this study was 227 (75.67%) (70.81-80.52 at 95% Confidence Interval) among whom only 35 (15.41%, CI 14.8-16.1%) were regular users. The major factors encouraging sunscreen use were to avoid tanning of the skin 89 (39.20%) followed by sunburn 83 (36.56%). Among 300 students, 130 (90.90%) female students used sunscreen as compared to 157 (61.78%) male students. Hence, the use of sunscreen was more prevalent among female students (Table 1).

**Table 1:** Demographic data of the participants (n = 300).

Character	Total n (%)	Sunscreen users
**Gender**		
Male	157 (52.3)	97 (61.78)
Female	143 (47.7)	130 (90.90)
**Academic year**		
1^st^ year	83 (27.67)	75 (90.36)
2^nd^ year	84 (28)	64 (76.19)
3^rd^ year	65 (21.67)	38 (58.46)
4^th^ year	68 (22.66)	50 (73.52)
**Fitzpatrick photo skin type**		
I-III	5 (1.67)	1 (20)
IV	91 (30.33)	43 (47.25)
V-VI	204 (68)	183 (89.70)

Among the female users, the most important criteria for choosing sunscreen was the packaging 36 (15.85%) and the influence from the family and friends 34 (14.97%) whereas in males, it was promotion, advertisement, and brand reputation 30 (13.21%).

The age of participants ranged from 19-26 years (mean age of 21.22±1.95) among them majority 204 (68%) have Fitzpatrick skin type V-VI (Table 1).

**Figure 1:**
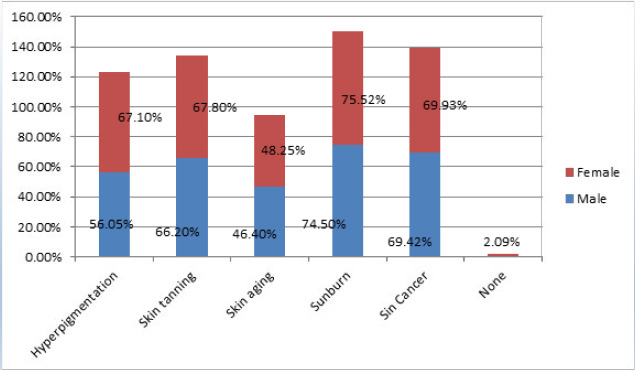
Damage caused by unprotected sun exposure.

Most students 268 (89.34%) knew hazards of unprotected sun exposure (Figure 1) and 181 (60.44%) knew the beneficial effects of regular sunscreen use (Table 2).

**Table 2:** Knowledge about sun exposure and the effects of sunscreen.

Questions	n (%)
Unprotected sun exposure is harmful.	290 (96.66)
Solar radiation is a major cause of skin cancer.	274 (91.33)
Person with dark skin also needs to use sunscreen	245 (81.66)
The use of sunscreen prevents skin cancer	181 (60.33)
Sunscreen reverses skin aging	89 (29.66)
It is necessary to use sunscreen even on a cloudy day	148 (49.33)

Among the non-users, the main reason for not using the sunscreen was forgetting to apply and the greasy feeling after its application. Among the sunscreen users, 109 (48.01%) applied immediately before sun exposure and 115 (50.66%) applied 30 mins before sun exposure. The majority 144 (63.43%) applied sunscreen only on the face. Among 72 (31.71%) who considered reapplication, only 6 (8.33%) reapplied every 2 hours, and 55 (76.38%) reapplied only after water exposure. The majority 155 (68.2%) of the sunscreen users knew the Sun Protection Factor (SPF) value and sunscreen with SPF 30 was the most preferred one 108 (47.57%). Greasy feeling 73 (100%) was the reason for avoiding sunscreen use amongst the non-users 73 (24.34%) and cosmetic sunscreen 151 (66.51%) was preferred more than the medicated ones 76 (33.48%) among the users.

**Table 3:** Additional sun protection behavior among the respondents (multiple responses) (n = 300).

Characteristics	Male n (%)	Female n (%)
Use additional sun protection methods (Umbrellas, full sleeves, hats etc.)	51 (17)	70 (23.33)
Frequency of sunscreen use	54 (18)	79 (26.33)
Change to more SPF value sunscreen	31 (10.33)	48 (16)
Amount of sunscreen	6 (2)	12 (4)
No additional sun protection behavior	80 (26.67)	75 (25)

## DISCUSSION

The prevalence of regular sunscreen use among medical undergraduate students of BPKIHS was 15.41% which is lower than the prevalence of regular users from western world.^6^ However, the overall prevalence of sunscreen users in this study was 75.67%, which is considerably higher than the prevalence of sunscreen use among medical undergraduate students in Jammu and Kashmir^7^ which was 48.88%, and a population based study conducted in Southern Brazil where the prevalence of sunscreen use was 60.8%.^8^ The most important criteria for choosing the sunscreen in this study were based on promotion, advertisement (38.32%) and packaging (28.18%). This could be because the media channels such as magazines and the leverage of mass media can sway how the public perceives information and can grab consumer's attention through advertisements and sales promotion.

In this study, the most important reason for avoiding sunscreen use among the non-users was their forgetting nature, followed by the greasy feeling after its application, whereas the major factor that influenced sunscreen use among the users was to avoid skin tanning (39.20%). This contradicts findings from studies conducted in western countries that having a tan is more physically attractive^9,10^ and that tanner people are better looking and attractive. This could be because of the differences in cultural background and the beauty standards, and the societal norms followed in our context that fairer people are more appreciative. This further contradicts the finding of this study that one of the most common adverse effects due to sun exposure is tanning (16.42%).

This study shows that 89.34% of participants correctly answered questions related to knowledge regarding hazards of unprotected sun exposure which supports the findings of studies conducted in Australia^11^ and the Unites States of America^12^ that showed 80% and 89% of the participants being competent regarding knowledge of dangers of sun exposure. This is entirely plausible that the medical students have had specific learning on hazards of unprotected sun exposure within their course, and their knowledge should expectedly be higher. However, female students were knowledgeable about the damages caused by unprotected sun exposure (55.11%) and also were more extensive sunscreen users (90.90%) than males (61.78%). Females in this study were more concerned regarding adopting additional sun protection behavior than males. This is similar to the results of other studies.^13-15^ This could be because females receive more counseling for beauty and makeup; they have more information about taking care of sunlight and protect themselves better.

However, in this study, only 60.44% had adequate knowledge regarding the beneficial effects of sunscreen use, whereas, studies reveal that in western countries knowledge regarding benefits of sunscreens is high.^16^ This could be because of the extensive public health campaign carried out in western countries due to the high prevalence of skin cancer.^2^ Additionally, in our context, sunscreens are viewed as cosmetics and are pricy, thus are used occasionally or seldom in general. In this study, the participants exhibited poor practice regarding application time and reapplication of the sunscreen which is contradictory to other studies.^17-19^ This exhibits the dearth of quality education and incomplete directions for effective sunscreen usage.

Only the medical undergraduate students were included in the study. This group falls under an educated group having a greater interest and knowledge than the general Nepalese population. Thus, the result of this study may not be generalizable to the general population of Nepal. The data is selfreported depending on the respondent's honesty and ability to recall, thus are subjected to recall bias.

## CONCLUSIONS

The prevalence of sunscreen use among medical undergraduate students from our study was high as compared to other studies; however, the regular users were less. Greasy feeling was the leading cause for avoiding sunscreen use. Adequate knowledge regarding the beneficial effects of sunscreen use was not satisfactory and a significant number of participants had misperceptions regarding effects of sunscreen. The results of this study reveal the need for proper medical education and future campaigns that focuses on the importance of sunscreen and the correct methods to apply them.
